# Strain-specific differences in the response to egg-derived versus recombinant protein influenza vaccines

**DOI:** 10.64898/2026.02.23.707528

**Published:** 2026-02-23

**Authors:** Andrea N Loes, Rosario AL Tarabi, Shuk H Li, Reilly K Atkinson, John Huddleston, Caroline Kikawa, Tachianna Griffths, Elizabeth M Drapeau, Sook-San Wong, Samuel MS Cheng, Nancy HL Leung, Sarah Cobey, Benjamin J Cowling, Trevor Bedford, Scott E Hensley, Jesse D Bloom

## Abstract

The 2023/2024 influenza vaccine included an updated H1N1 component designed to better match a new clade of H1N1 that had multiple mutations in antigenic epitopes of hemagglutinin. Despite this update, the vaccine trended towards being less effective against the vaccine-matched H1N1 clade than the parental H1N1 clade lacking the new antigenic mutations. Here we measure neutralization titers of serum antibodies from individuals who had received either a recombinant protein or an egg-derived vaccine against a set of viruses with hemagglutinins from 58 H1N1 strains representative of the diversity during the 2023/2024 season. We find that egg-derived vaccine recipients, but not recombinant protein vaccine recipients, had a relatively lower boost in neutralizing titers to the new clade that the updated vaccine was designed to target. We suggest that the difference in the extent that the egg-derived versus recombinant protein vaccines boost neutralizing titers to the new H1N1 clade is because the seed strain for the egg-derived vaccine strain had acquired a reversion of a key antigenic mutation (K142R) present in that clade. Our results show how egg-derived versus recombinant protein vaccines can elicit different relative titer boosts against different subsets of viral strains, a phenomenon that could impact vaccine effectiveness.

## Full Text Availability

The license terms selected by the author(s) for this preprint version do not permit archiving in PMC. The full text is available from the preprint server.

