# Supplementary material for: Strain-specific differences in the response to egg-derived versus recombinant protein influenza vaccines
Source: bioRxiv. 2026 Apr 20:2026.02.23.707528. Originally published 2026 Feb 23. Preprint. [Version 2] doi: 10.64898/2026.02.23.707528 (PMC12970319; doi:10.64898/2026.02.23.707528)
Supplement: Supplement 1 [file NIHPP2026.02.23.707528v2-supplement-1.pdf]

bioRxiv preprint doi: https://doi.org/10.64898/2026.02.23.707528; this version posted April 20, 2026. The copyright holder for this preprint (which was not certified by peer review) is the author/funder, who has granted bioRxiv a license to display the preprint in perpetuity. It is made available under aCC-BY 4.0 International license.

Supplemental Figures

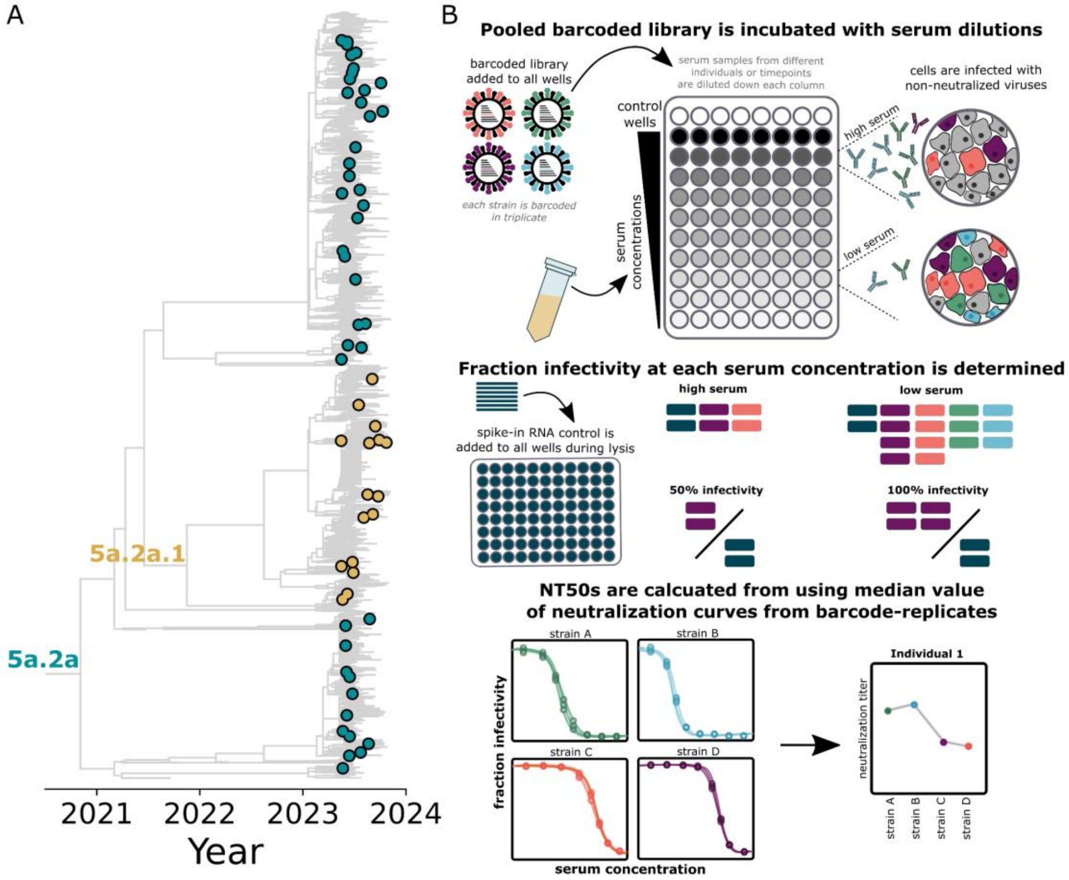

Supplemental Figure 1. Strains selected for the sequencing-based neutralization assay represent the diversity of H1N1 viruses circulating in the fall of 2023. A) Phylogenetic tree of strains selected representing top haplotypes circulating in fall of 2023 from https://nextstrain.org/seasonal-flu/h1n1pdm/ha/6m@2023-11-16. Strains selected for inclusion in the library are indicated with circles. Strains within the 5a.2a.1 clade are labelled in gold and other 5a.2a clade strains are labelled in teal. B) Schematic of the sequencing-based neutralization assays. Briefly, pooled barcoded viruses are incubated with serum dilutions for 1 hr, and then cells are added such that non-neutralized viruses can infect and amplify barcoded HA vRNA segments. At 16 hrs, a barcoded RNA spike-in control is added at the same concentration to each well, such that the fraction infectivity of each barcoded strain can be estimated from the fraction of that barcode to the fraction of the barcoded RNA spike-in control in a given well, compared to the fraction at which that barcode is represented in the no-serum control wells. Curves are then fit to the fraction infectivity measurements to calculate a neutralization titer at 50% infectivity (or NT50).

bioRxiv preprint doi: https://doi.org/10.64898/2026.02.23.707528; this version posted April 20, 2026. The copyright holder for this preprint (which was not certified by peer review) is the author/funder, who has granted bioRxiv a license to display the preprint in perpetuity. It is made available under aCC-BY 4.0 International license.

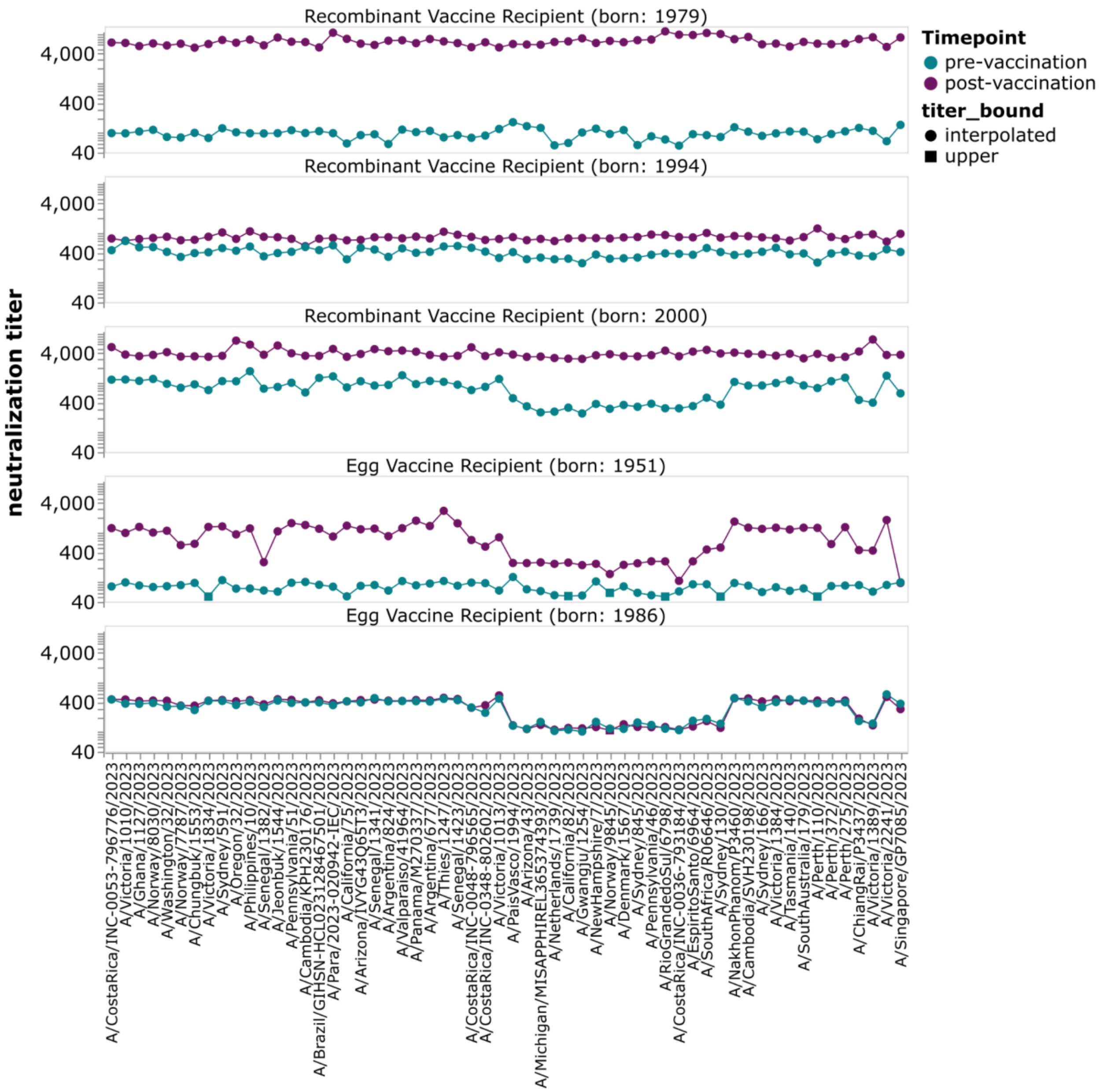

Supplemental Figure 2. Heterogeneity among individuals in response to vaccination and neutralizing titers to recent strains. Neutralization titers against the library of 2023 H1N1 strains for pre- and post-vaccination sera from some example individuals who received either the recombinant protein or egg-derived vaccine. The points represent the titer against each strain. Strains are grouped phylogenetically along the x-axis. Some calculated titers were outside the range of dilutions tested (titer_bound); NT50s shown as squares, indicated as being at the upper bound, were below the lowest dilution tested.

bioRxiv preprint doi: https://doi.org/10.64898/2026.02.23.707528; this version posted April 20, 2026. The copyright holder for this preprint (which was not certified by peer review) is the author/funder, who has granted bioRxiv a license to display the preprint in perpetuity. It is made available under aCC-BY 4.0 International license.

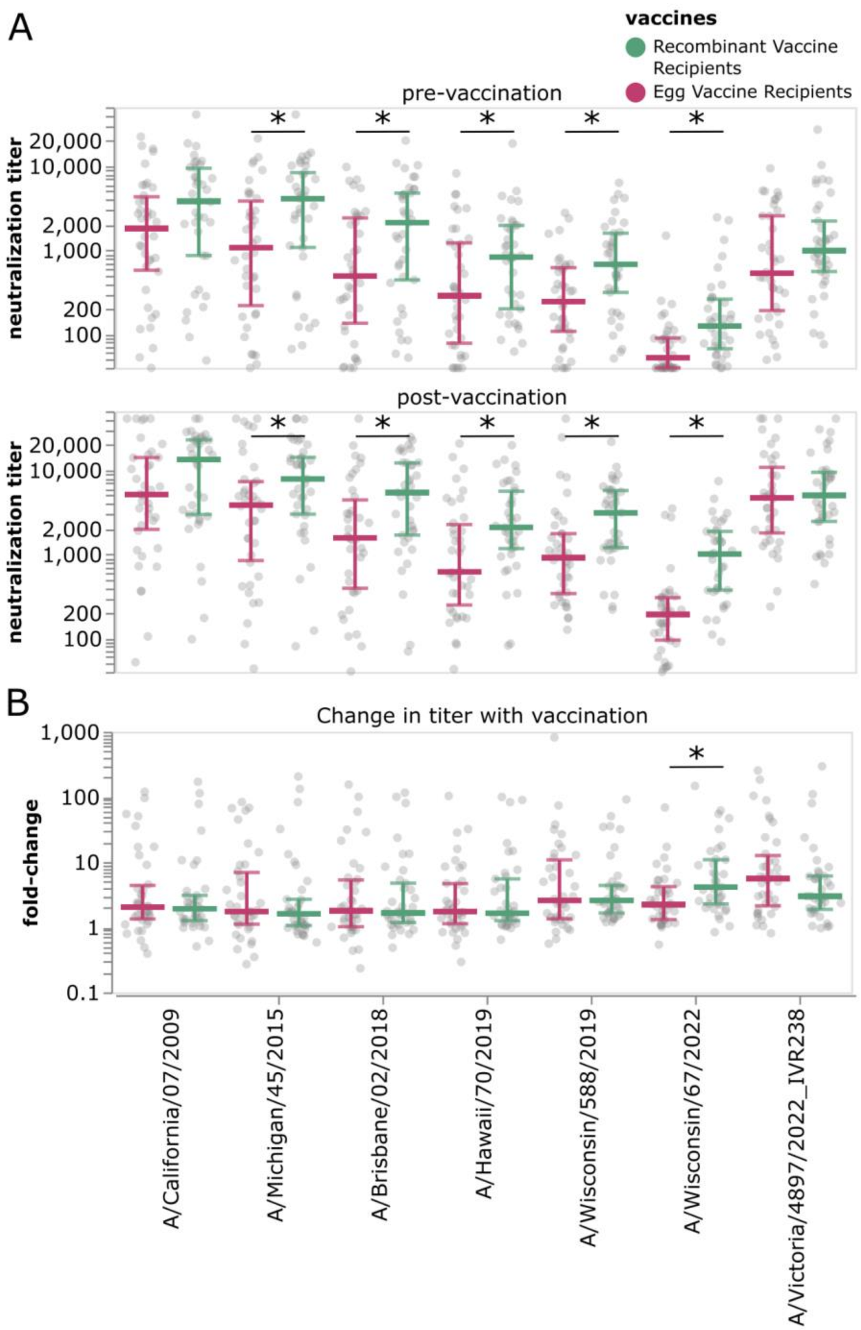

Supplemental Figure 3. Neutralizing antibody titers to prior vaccine strains. A) Neutralization titers pre- and post- vaccination against previous seasons' vaccine strains. B) Fold-change in titer against previous vaccine strains after vaccination. Grey points show the measured titer or fold-change for a single individual in each group. Colored horizontal lines represent the median titer for all individuals in a given group against that vaccine strain. Error bars show the interquartile range for that group. Strains with a significant difference in titer or fold-change between groups as assessed by Mann-Whitney U test are indicated with an asterisk.

bioRxiv preprint doi: https://doi.org/10.64898/2026.02.23.707528; this version posted April 20, 2026. The copyright holder for this preprint (which was not certified by peer review) is the author/funder, who has granted bioRxiv a license to display the preprint in perpetuity. It is made available under aCC-BY 4.0 International license.

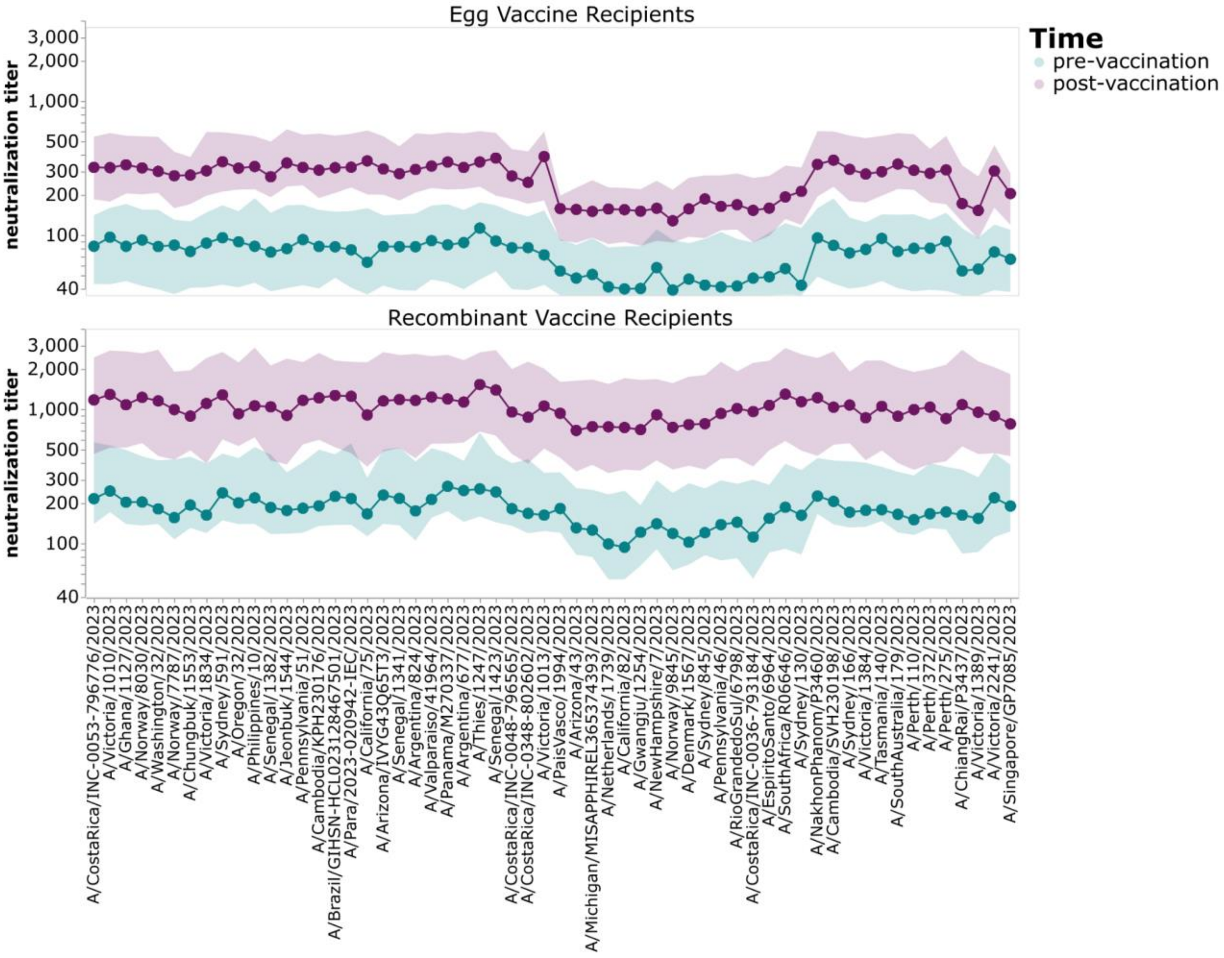

Supplemental Figure 4. Pre- and post-vaccination titers against a set of H1N1 strains that circulated in 2023/2024 for adults born between 1976 and 2001 who received egg-derived or recombinant protein vaccines. This figure shows data comparable to Figure 2 except it includes only the individuals in the cohorts that were born between 1976 and 2001.

bioRxiv preprint doi: https://doi.org/10.64898/2026.02.23.707528; this version posted April 20, 2026. The copyright holder for this preprint (which was not certified by peer review) is the author/funder, who has granted bioRxiv a license to display the preprint in perpetuity. It is made available under aCC-BY 4.0 International license.

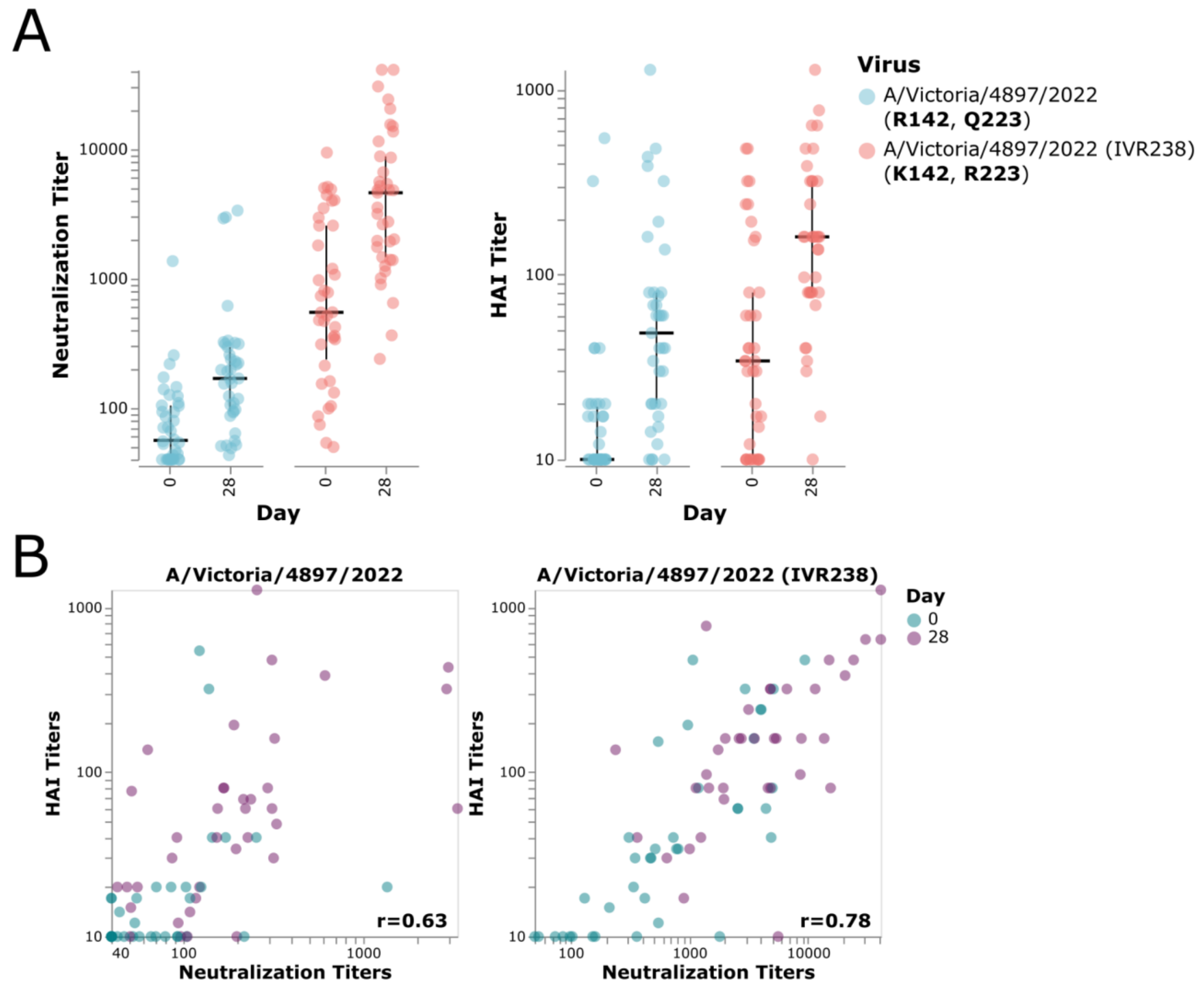

Supplemental Figure 5. Comparison of hemagglutinin inhibition titers (HAI) and sequencing based-neutralization titers against the A/Victoria/4897/2022 (IVR238) vaccine strain and A/Victoria/4897/2022 strain without egg-adaptive mutations (R142K, Q223R) for individuals who received the egg-derived vaccine. A) Titers collected with sequencing-based neutralization assay and with HAI assay against A/Victoria/4897/2022 and A/Victoria/4897/2022(IVR-238) show similar trends. Points show measurements for each individual against each strain at day 0 or day 28, respectively. Horizontal lines show median values for that group and vertical bars indicate the interquartile range. B) Titers at day 0 and day 28 collected by either HAI or with sequencing-based neutralization assay are well correlated. Points show measurements for each individual against each strain at day 0 or day 28, respectively and are colored by the day sera was collected. Pearson correlations (r) are indicated.

bioRxiv preprint doi: https://doi.org/10.64898/2026.02.23.707528; this version posted April 20, 2026. The copyright holder for this preprint (which was not certified by peer review) is the author/funder, who has granted bioRxiv a license to display the preprint in perpetuity. It is made available under aCC-BY 4.0 International license.

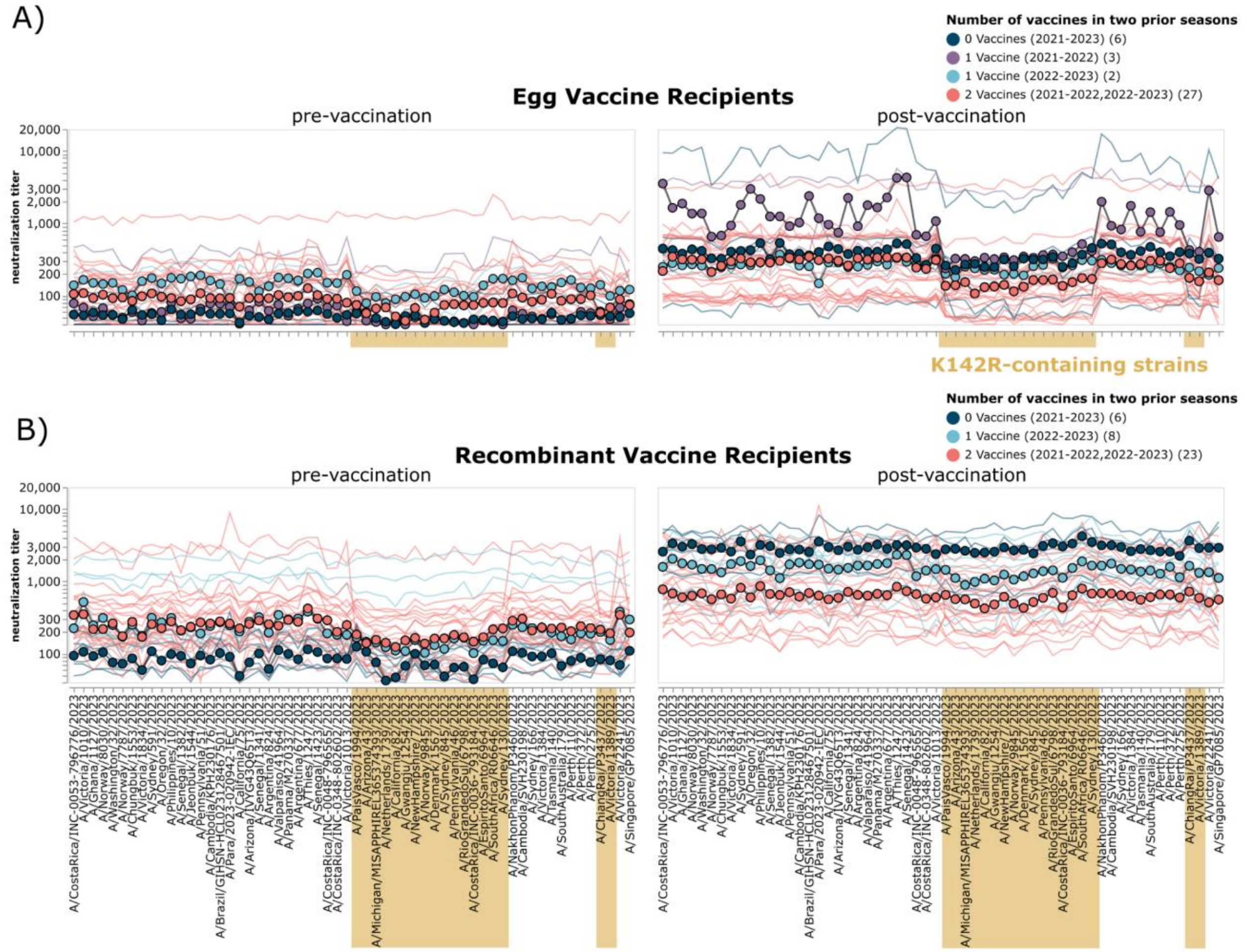

Supplemental Figure 6. Vaccination history in the two seasons preceding 2023/2024 is not obviously related to the relatively lower titers to K142R-containing strains. A) Neutralization titer profiles for pre- and post-vaccination sera from egg-derived vaccine recipients. B) Neutralization titer profiles for pre- and post-vaccination sera from recombinant vaccine recipients. Thin lines show a neutralization titer profile for a given individual. Each point represents the median neutralization titer across all sera in that group for that strain. Strains are organized phylogenetically on the x-axis. Strains containing K142R are indicated in gold. A shared x-axis is used for both panels and a colored bar below panel A is provided to facilitate easier association with labeled strains. Recombinant vaccine recipient group with 2 vaccines in 2021-2023 seasons includes individuals with and without vaccines in 2020-2021 season. Vaccination status for egg-derived vaccine recipients in 2020-2021 season is unknown.

bioRxiv preprint doi: https://doi.org/10.64898/2026.02.23.707528; this version posted April 20, 2026. The copyright holder for this preprint (which was not certified by peer review) is the author/funder, who has granted bioRxiv a license to display the preprint in perpetuity. It is made available under aCC-BY 4.0 International license.

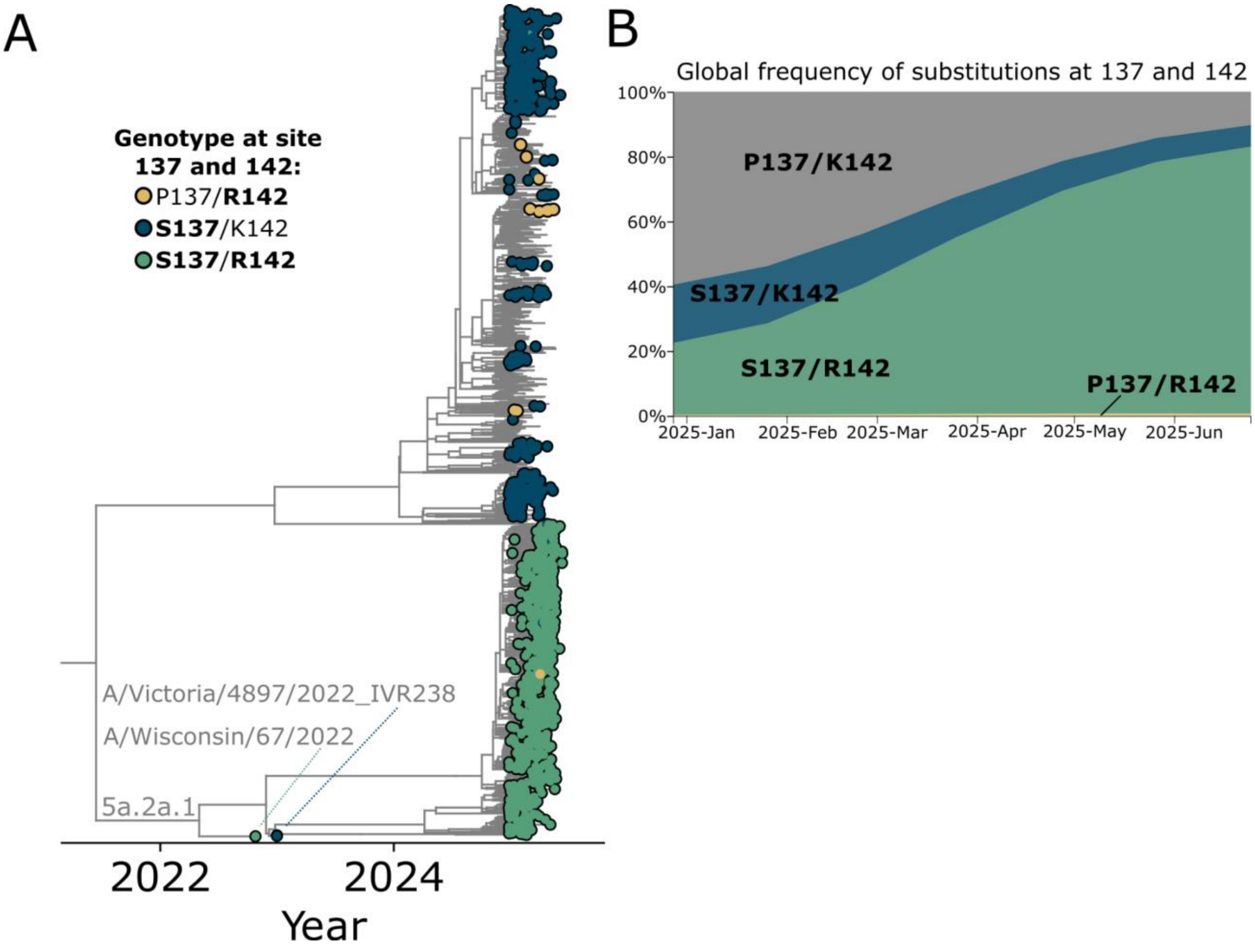

Supplemental Figure 7. Global frequency of mutations identified to have antigenic effects on pre- and post-vaccination sera first 6 months of 2025 A) A 6-month H1 phylogeny adapted from https://nextstrain.org/seasonal-flu/h1n1pdm/ha/6m@2025-06-26 from Nextstrain[41,42], showing an increase in strains containing P137S and K142R. Strains containing the K142R mutation only are indicated with yellow circles at the tips, while strains containing P137S only are indicated with navy circles. Strains that contain both P137S and K142R are indicated with green circles. B) The global frequency over time of strains containing mutations P137S and/or K142R.
